# Roma Health: An Overview of Communicable Diseases in Eastern and Central Europe

**DOI:** 10.3390/ijerph17207632

**Published:** 2020-10-20

**Authors:** Kabir Tombat, Jitse P. van Dijk

**Affiliations:** 1 Department of Community and Occupational Medicine, University Medical Center Groningen, University of Groningen, Ant. Deusinglaan 1, 9713 AV Groningen, The Netherlands; j.p.van.dijk@umcg.nl; 2 Graduate School Kosice Institute for Society and Health, Medical Faculty, P.J. Safarik University, Tr. SNP 1, 040 01 Kosice, Slovakia; 3 Olomouc University Society and Health Institute, Palacky University, 771 11 Olomouc, Czech Republic

**Keywords:** Roma, communicable diseases, Central and Eastern Europe, review

## Abstract

The Roma are Europe’s largest minority. They are also one of its most disadvantaged, with low levels of education and health and high levels of poverty. Research on Roma health often reveals higher burdens of disease in the communities studied. This paper aims to review the literature on communicable diseases among Roma across Eastern and Central Europe. A PubMed search was carried out for communicable diseases among Roma in these parts of Europe, specifically in Romania, Bulgaria, Hungary, Serbia, Slovakia, the Czech Republic and North Macedonia. The papers were then screened for relevance and utility. Nineteen papers were selected for review; most of them from Slovakia. Roma continue to have a higher prevalence of communicable diseases and are at higher risk of infection than the majority populations of the countries they live in. Roma children in particular have a particularly high prevalence of parasitic disease. However, these differences in disease prevalence are not present across all diseases and all populations. For example, when Roma are compared to non-Roma living in close proximity to them, these differences are often no longer significant.

## 1. Introduction

The Romani, or Roma are the largest transnational minority in Europe. Through linguistic, anthropological and more recently genetic mapping, their roots can be traced to original nomadic communities in north-west India [1]. They began their migration westwards between the 6th to 10th century, with groups settling along the way, finally entering Europe in the 12th century. Roma communities across Europe and Central Asia gradually formed diverse endogamous sub-groupings, still retaining large parts of their language and culture [1,2]. In Europe alone they now number around 11 million, the vast majority living in Eastern and Central Europe (Table S1). 

The history of Roma people in Europe is marked by discrimination and persecution. In Romania, they were enslaved [3]; in Britain they were declared criminals and had to choose between exile or death [4]. During Nazi rule, they became the victims of genocide, which the Roma call “Porajmos” [5]. In Eastern Europe, Roma children were taken from their parents in an attempt at assimilation. More recently, in the Czech Republic, Roma women were unwittingly sterilised even after the turn of the millennium [6]. Even now, in 2020, in the midst of the coronavirus pandemic, a few Roma settlements in Slovakia and Bulgaria are being subjected to disproportionately high levels of surveillance and policing [7]. These are just a few examples of discrimination and persecution which the Roma have endured and continue to face. Though commonly thought of as itinerant, most Roma are actually settled, partly on account of forced assimilation policies. 

By all accounts, the Roma are extremely disadvantaged. Most people identifying as Roma live in informal settlements, often with facilities which are far below the national standards of the countries they are settled in [8]. Roma are also routinely found to have worse social, economic and health indicators than their non-Roma counterparts. Ninety percent of Roma live below national poverty lines. Less than one third are in paid employment. Only 15 per cent of Roma have completed high school, and 45 per cent of Roma households lack proper sanitation [8,9]. Roma children are only 34 to 45 per cent as likely to be vaccinated as non-Roma, and they routinely face barriers in accessing healthcare [10,11,12]. 

In 2005, nine Central and Southern EU countries—Bulgaria, Croatia, the Czech Republic, Hungary, North Macedonia, Romania, Serbia, Montenegro and Slovakia—along with several international organisations, launched the *Decade of Roma Inclusion 2005–2015*, committing to allocate resources with the aim of integration and ending discrimination and poverty of Roma communities. This was followed by the *Roma Integration 2020 Project*, with similar goals [13]. There are numerous papers examining the prevalence of specific diseases in certain Roma communities. This paper aims to examine the occurrence of communicable diseases among Roma across Eastern and Central Europe. 

## 2. Methods

### 2.1. Sample

Publications were selected based on a PubMed search, starting in 2005 and ending July 2020. The literature selected describes the occurrence of communicable disease among the Roma in Eastern European countries. These were limited to EU member states or states in the accession process with sizeable Roma populations. EU member states and those in the accession process were chosen for ease of comparability based on the similarity of their legal systems, policies and institutions. The Roma population had to be sizeable, i.e., being defined as at least 5 per cent of the total population and/or amounting to at least 200,000 persons. The size of the Roma population was also taken into account for compatibility purposes. For example, Russia and Ukraine are currently not in the process of EU accession and were therefore excluded, despite having sizeable Roma populations. The countries of study selected were Romania, Bulgaria, Hungary, Serbia, Slovakia, the Czech Republic and North Macedonia (Table S1). Including countries outside of these parameters was beyond the scope of this short study, but they nevertheless warrant further research. Turkey, which has an estimated Roma population of 2.75 million, was excluded since EU accession negotiations have reached a virtual standstill. Spain, France and the UK, which are EU member states with sizeable Roma populations outside Central and Eastern Europe, were also intentionally excluded.

The following search strategy was employed. The search string detailed the target population, i.e., the Roma; the geographical focus, i.e., the seven specified countries; and lastly communicable diseases (Supplementary Material S1). Text word terms were used for “Roma” and its more commonly-used exonyms and endonyms, such as Romani, Gypsy, Romany, Sinti. Certain terms carrying a geographical marker not relevant to the intended geographical area of study, such as Gitano, which is one of the terms used for Roma people in Spain, were intentionally excluded. Gypsy, Sinti and their various iterations were included, despite being geographically inappropriate, owing to their generic usage for the Roma in English publications.

Text word terms were employed for the countries selected to make the search as broad as possible. Using the same search string without specifying countries yielded almost 1177 results, many of which focused on orthopaedic papers dealing with range of motion (ROM), rod outer-segment membrane protein, or non-included countries such as Greece and Spain.

For communicable diseases, a combination of MeSH and text word terms were used for the various communicable diseases specifically, including but not limited to listing all the diseases mentioned on the WHO Regional Office for Europe’s pages on communicable diseases [14], as well as umbrella terms such as *sexually transmitted diseases*. This was done to make the search as broad as possible.

### 2.2. Reporting

The Decade of Roma Inclusion was launched in 2005, when several European governments committed to improve the conditions of their Roma minorities. This search yielded 96 results. Other filters such as language and “human” or “other animal” were not used, since the paper classification was often incomplete. Out of the 96 papers, all of which pertained to humans, only 85 were marked as “human”.

**Figure 1:**
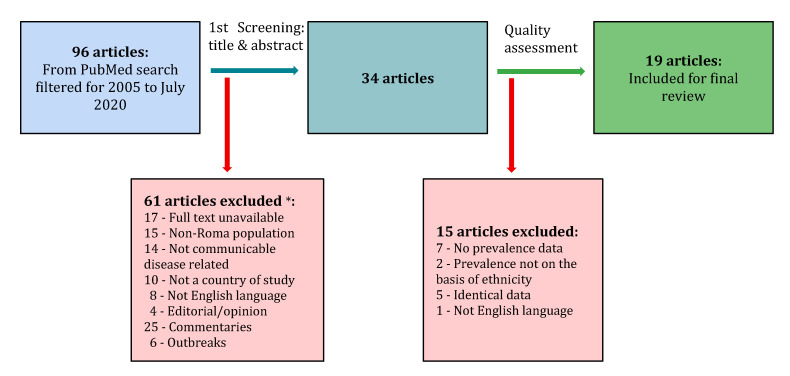
Flowchart showing steps for inclusion/exclusion. * Reasons for exclusions may overlap.

The titles and abstracts were then preliminarily screened for relevance, as shown in Figure 1. Papers were included if they pertained to prevalence of any communicable diseases among the Roma community within any of the specified seven countries. Papers exclusively describing outbreaks were therefore excluded. The papers had to have been published in English and had to have full text availability. Opinion pieces, editorials and commentaries were excluded. If titles and abstracts were ambiguous in terms of the above criteria, the full text was scanned as well. This resulted in 35 papers which were selected for closer examination. Papers were further excluded, if on scanning the full texts they were found not to fulfil the inclusion criteria stated in the initial screening, such as describing prevalence data. This resulted in papers being excluded for not reporting data on the basis of ethnicity and not reporting specific disease occurrence. Further exclusions were made for multiple papers based on the same databases, for which the population and prevalence data were identical. This was the case of five papers from the HepaMeta team in Slovakia. In this case, only the first published paper was included.

A quality assessment was then carried out. This relied on the Joanna Briggs Critical Appraisal Checklist for Prevalence Studies [15]. This was further quantified by assigning a numerical value of 1 to every yes. 

## 3. Results

Ultimately, 19 papers were selected for review, as illustrated in Figure 1 and Table 1. The included papers studied mostly high-risk sub-populations of Roma. All the papers involved Roma in settlements with substandard living conditions. Twelve of the 19 papers are from Slovakia. The Czech Republic and North Macedonia are not represented in the final selection of papers to be studied. Many papers are authored by the same research teams, for example, six papers from Slovakia are by the HepaMeta team and rely on the same database, which examines a cross-section of Roma and non-Roma in Košice in eastern Slovakia.

### 3.1. Viral Disease

The prevalence of human immunodeficiency virus (HIV) among pregnant Roma women (*n* = 862) was 0.6 per cent vs. 0.1 per cent among “white” women (*n* = 10,192) in a pilot prevention of mother-to-child HIV transmission programme in south-east Romania. The study was carried out in Constanta county, which was known for an HIV outbreak [16]. Young Roma men in Bulgaria (*n* = 405) had an HIV prevalence of 0.5 per cent [20]. In Budapest, Hungary, however, neither of the two populations of Roma surveyed had HIV, despite one group being made up of injecting drug users (IDUs) [18,19].

Hepatitis infection prevalence in Table 2 details lifetime infection, and in a study of 64 Hungarian volunteers from a predominantly Roma neighbourhood of Budapest, 26.0 per cent of the Roma population (*n* = 50) had resolved hepatitis B virus (HBV) infections vs. 28.6 per cent of non-Roma (*n* = 14). None, however, had an active infection. Roma had higher infection rates for hepatitis A virus (HAV) and hepatitis C virus (HCV), at 80.0 per cent and 26.0 per cent, respectively, vs. 42.9 per cent and 14.3 per cent among non-Roma. Ten per cent of Roma had antibodies against HAV, HBV and HCV. Within this population, 45.0 per cent had used illicit drugs, and 28.0 per cent had injected drugs [18]. In eastern Slovakia, 12.5 per cent of the Roma surveyed (*n* = 441) had an active HBV infection and 40.4 per cent had a resolved infection. However, only 0.7 per cent of Roma tested positive for HCV [22]. A subpopulation of the same sample was tested for hepatitis E virus (HEV), and 21.5 per cent of Roma tested positive vs. 7.2 per cent of non-Roma [31].

A Romanian cervical cancer screening study found human papillomavirus (HPV) prevalence among Roma (*n* = 124) to be 6.5 per cent vs. 15.5 per cent of women identifying as Romanian (*n* = 1615). In fact, the only minority with a lower prevalence at 4.2 per cent consisted of women identifying as Ukrainian (*n* = 24) [32]. 

### 3.2. Parasitic Disease

Parasitic disease in children was the focus of six of the ten studies covering parasitic diseases. Except for one paper describing an 8.7 per cent prevalence of Trichomonas among young Roma men in Sofia, Bulgaria [17], all the other papers cover Roma in Slovakia (Table 3). 

The prevalence of microsporidia in the stool samples of clinically-healthy Roma children (*n* = 72) from settlements in eastern Slovakia was 30.6 per cent. Of these, the prevalence of *Enterocytozoon bieneusi* was 4.2 per cent and *Encephalitozoon cuniculi* 26.4 per cent. The highest prevalence was found in boys aged 6–9 years (*n* = 11) at 45.5 per cent, and the risk of infection was 1.8 times higher in the group of boys [21].

Among children aged 1–2 years hospitalised at the Institute for Child and Youth Health Care of Vojvodina in Novi Sad, Serbia, 10.0 per cent of Roma children (*n* = 59) had parasitic skin disease (*Pediculus humanus capitis* and scabies) vs. 0.0 per cent of the non-Roma children (*n* = 59) [24].

The prevalence of cryptosporidium in the stool samples of clinically-healthy Roma children (*n* = 53) in eastern Slovakia was 11.3 per cent, whereas 0.0 per cent of the non-Roma children sampled (*n* = 50) tested positive. Roma babies less than one year old had the highest prevalence, at 22.7 per cent [25].

In the Košice region of eastern Slovakia, seropositivity to Toxocara was 22.1 per cent among Roma (*n* = 429) compared to 1.0 per cent among non-Roma (*n* = 394). Increasing age (Odds Ratio (OR) 2.512, 95% Confidence Interval (CI) 1.477–4.271) and the lack of household hygiene facilities (OR 2.512, 95% CI 1.477–4.271) were both strong risk factors for seropositivity [26]. In another Slovakian study, prevalence among Roma children (*n* = 67) was 40.3 per cent vs. 2.3 per cent among non-Roma children (*n* = 44) [34].

The prevalence of helminthic infections in hospitalised and non-hospitalised children in the Prešov and Košice regions of eastern Slovakia was 25.8 per cent among Roma children (*n* = 275) vs. 0.7 per cent among non-Roma children (*n* = 150). A single species, *Ascaris lumbricoides,* accounted for 87.5 per cent of all helminthic infections. The age groups 3–5 years (*n* = 64) and 6–10 years (*n* = 57) among Roma had the highest prevalence of these infections at 31.3 per cent and 30.2 per cent, respectively [27]. In Medzev, 36 km west of Košice, 85 per cent of Roma children (*n* = 60) had helminthic infections vs. 23.8 per cent of non-Roma children (*n* = 21). Seroprevalence for *Strongyloides stercoralis* specifically was 33.3 per cent in Roma children vs. 23.8 per cent in non-Roma [28]. In the HepaMeta population, seropositivity for Trichinella or Echinococcus was 0.5 per cent and 0.2 per cent among Roma tested (*n* = 429). However, no significant difference was found with regard to non-Roma (*n* = 394) [29].

Prevalence of *Toxoplasma gondii* in the HepaMeta population in eastern Slovakia, determined *by* seroprevalence of *T. gondii* antibodies, was 45.0 per cent among Roma (*n* = 420) compared to 24.1 per cent among non-Roma (*n* = 386). Prevalence among non-Roma living in the vicinity of the Roma settlements (*n* = 158) was 30.4 per cent compared to 19.7 per cent among non-Roma outside this area (*n* = 228) [30]. In a group of Roma children from across Slovakia (*n* = 67), seroprevalence was 20.9 per cent vs. 7.1 per cent among non-Roma children (*n* = 42) [33].

### 3.3. Bacterial Disease

Papers on bacterial disease among the Roma all examine sexually transmitted diseases (Table 4). A study of young Roma men in Sofia, Bulgaria (*n* = 296) found that 21.7 per cent had at least one STD (trichomonas, chlamydia, gonorrhoea or syphilis) and that the rates of gonorrhoea and syphilis were 1807 and 312 times the national levels, respectively [17]. A later study by the same group of researchers examining a larger group of young Roma men found much lower rates of infection [20].

The prevalence of syphilis in a sample of volunteers tested at a health camp in a predominantly Roma neighbourhood of Budapest, Hungary was 1.8 per cent among Roma (*n* = 50) vs. 0.0 per cent among non-Roma (*n* = 14) [18]. A study by the same authors on IDUs in Budapest showed that 16.7 per cent of Roma IDUs (*n* = 42) were positive for either chlamydia or syphilis vs. 8.3 per cent of non-Roma IDUs *(n* = 144), while none of the IDU’s sampled tested positive for gonorrhoea [19].

The prevalence of *Chlamydia trachomatis* in the HepaMeta population in eastern Slovakia was 7.2 per cent among the Roma (*n* = 208) compared to 5.3 per cent among non-Roma (*n* = 132). However, this difference was not significant. Roma women (*n* = 142) had a prevalence of 8.5 per cent compared to 4.5 per cent among Roma men (*n* = 66). There was no difference in prevalence among non-Roma men (*n* = 75) and women (*n* = 57) [23].

## 4. Discussion

The aim of this paper was to review the literature on communicable diseases among Roma across Eastern and Central Europe. We found that Roma communities have disproportionately high prevalence of communicable diseases, and are identified as being at high risk of infection throughout these parts of Europe. 

Studies on communicable diseases among Roma appear to originate primarily from Slovakia. Romania, which has the highest population of Roma in the EU, has surprisingly little research published on them. In this review only two papers on Romanian Roma met the selection criteria. The reasons for this disparity need to be examined. This review was only concerned with papers written in English, so it is possible that more information is available in the national languages. 

The papers reviewed all involved segregated Roma, i.e., those living in settlements. Though this might seem to be a limitation, it is known that most Roma in Europe identifying as Roma live predominantly in informal settlements [8]. Furthermore, data collection along ethnic lines remains a contentious issue [35]. Additionally, most integrated Roma no longer identify as Roma, or might not even know of their Roma heritage [36]. As a result, there is a lack of usable data on Roma living outside the settlements.

This review indicates that Roma sometimes do not have a higher prevalence of communicable diseases. No significant difference between Roma and non-Roma was found in chlamydia cases in the HepaMeta population in eastern Slovakia. In fact, Roma men had fewer cases of chlamydia than either non-Roma men or women. Nevertheless, the authors of this review tentatively state that Roma are at a higher risk of contracting chlamydia, and are more likely to suffer from adverse effects of infection because of the barriers to healthcare which they face [23]. Drawing from the same HepaMeta population, seropositivity for Trichinella or Echinococcus showed no statistical differences between Roma and non-Roma [29]. Among a group of IDUs in Budapest, neither the Roma nor the non-Roma IDUs had HIV [19]. 

Non-Roma living in close proximity to Roma do not have very different prevalence of communicable diseases than the Roma. In fact, Roma may even have lower rates of disease. This is evidenced by some surprising findings. Non-Roma residents of a predominantly Roma neighbourhood of Budapest had higher HBV rates than their Roma neighbours, while neither of the groups had any cases of HIV [18]. A HepaMeta subpopulation in Slovakia showed higher rates of *T. gondii* among non-Roma living in close proximity to Roma settlements [30].

Roma have a relatively high prevalence of communicable diseases overall. Notwithstanding the instances mentioned above, Roma have a higher occurrence of communicable diseases than non-Roma. The most common reasons hypothesised by authors for the higher rates of disease are lack of water, poor sanitation and hygiene, crowded living spaces, high-risk sexual behaviours, and exposure to animals and waste [16,17,18,19,20,21,22,23,24,25,26,27,28,29,30,31,32,33,34].

Several studies found that Roma, especially Roma children, have a particularly high prevalence of parasites. This is especially troubling, given that Roma children have been found to suffer from significantly higher levels of morbidity than their non-Roma peers [24], and because parasites such as microsporidia and toxocara can be life-threatening. Many of these are diseases of poverty, so they can potentially be treated and prevented, but they may result in significant morbidity if not managed.

Some studies found that Roma have high rates of sexually transmitted diseases. In Sofia, for example, the prevalence of gonorrhoea and syphilis was many hundred times the national levels [17]. On the other hand, despite repeated studies describing high-risk sexual behaviour, the prevalence of STDs is sometimes not very high [20,23]. Amirkhanian et al. hypothesize that this is probably due to the social insularity of the groups [20]. This might also explain the lower rates of HPV among Roma in Romania [32].

### 4.1. Strengths and Limitations

This review is the first to attempt an examination of the prevalence of communicable diseases among Roma across Eastern and Central Europe. However, some limitations should be mentioned. Papers were not screened very stringently for quality, given the dearth of research in this area. While this enabled the review to include a much broader range of papers, it also meant however that not all the papers included lent themselves to rigorous statistical analysis. Papers written in languages other than English were not searched for or included, as a result of which useful data might be missing. Transnational, national and regional databases of health were not examined either. Finally, analyses of impacts, such as socio-cultural aspects of the community, could only be included in as far as they were analysed in the reviewed papers.

### 4.2. Implications

Firstly, the higher prevalence of disease within Roma settlements, along with many of the risk factors for infections, should be a cause for concern and action. The Roma minority represents an untapped repository of knowledge and skill, and a lot more resources should be devoted to removing barriers to their full participation in all areas of society. Secondly, and perhaps more concerning to many, there is the fact that as long as segregated Roma continue to exhibit a higher prevalence of disease, they remain a reservoir for neglected and immunisable diseases which could easily spill over into the general population. This has happened several times already in the case of measles [37,38]. Lastly, Roma communities that do not have a high disease burden also need attention. Once diseases enter these insular communities, they are likely to spread rapidly given the high infection potential associated with lack of infrastructure, poor hygiene and frequent high-risk behaviours [23].

## 5. Conclusions

Roma in Eastern and Central Europe continue to have a higher prevalence of communicable diseases than the majority populations of the countries they live in. Roma children in particular have a particularly high prevalence of parasitic disease. However, these differences in disease prevalence are not always present across diseases and Roma populations. In the case of HPV in Romania for example, Roma women have less than half the rate of the disease than non-Roma Romanian women. Additionally, when Roma are compared to non-Roma living in close proximity to them, these differences are often no longer significant. This does not change the reality that Roma communities continue to score lower on socio-economic indicators, have a disproportionately high incidence of communicable diseases, and have been found to be at high risk of infection. 

## Figures and Tables

**Table 1:** Papers selected for review.

First Author	Pub. Year	Country of Study	Quality Score 9-Point Scale *	Total Sample Size	No. of Roma within the Sample	Target Group
Cocu M [16]	2005	RO	7	11423	862	Pregnant women
Kabakchieva E [17]	2006	BG	8	296	296	Young Roma men
Gyarmathy VA [18]	2008	HU	6	64	50	Convenience sample of volunteers
Gyarmathy VA [19]	2009	HU	7	186	42	Injecting Drug Users
Amirkhanian YA [20]	2013	BG	8	405	405	Young Roma men
Halánová M [21]	2013	SK	5	72	72	Roma children 0–14 years
Veselíny E [22]	2014	SK	7	855	441	HepaMeta subpopulation ^+^
Halánová M [23]	2014	SK	6	340	208	HepaMeta subpopulation
Djurovic D [24]	2014	SRB	3	118	59	Hospitalised children 1–2 years
Hasajová A [25]	2014	SK	6	103	53	Children 0-14
Antolová D [26]	2015	SK	7	823	429	HepaMeta subpopulation
Pipiková J [27]	2017	SK	6	426	275	Children
Štrkolcová G [28]	2017	SK	5	81	60	Children 0–17
Antolová D [29]	2018	SK	7	823	429	HepaMeta subpopulation
Antolová D [30]	2018	SK	7	806	420	HepaMeta subpopulation
Halánová M [31]	2018	SK	7	264	195	HepaMeta subpopulation
Ilisiu MB [32]	2019	RO	8	2060	124	Women 18–68
Fecková M [33]	2020	SK	6	1536	67	Children
Fecková M [34]	2020	SK	6	1489	67	Children

* Quality score based on Joanna Briggs Critical Appraisal Checklist for Prevalence Studies; see Table S2. ^+^ Roma and non-Roma from settlement regions in addition to non-Roma from other regions. RO—Romania; BG—Bulgaria; HU—Hungary; SK—Slovakia; SRB—Serbia.

**Table 2:** Viral disease (per cent).

First Author	Pub. Year	Country	No. of Roma	Target Group	HIV	HSV *	Any Hepatitis **	HAV	HBV	HCV	HEV	HPV
Cocu M	2005	RO	862	Pregnant women	0.6							
					(0.1)							
Gyarmathy VA	2008	HU	50	Convenience sample of volunteers	0.0			80.0	26.0	26.0		
					(0.0)			(42.9)	(28.6)	(14.3)		
Gyarmathy VA	2009	HU	42	Injecting Drug Users	0.0	100.0	71.3					
					(0.0)	(56.5)	(45.1)					
Amirkhanian YA	2013	BG	405	Young Roma men	0.5							

Veselíny E	2014	SK	441	HepaMeta subpopulation					52.8	0.7		
	
Halánová M	2018	SK	195	HepaMeta subpopulation							21.5	
											(7.2)	
Ilisiu MB	2019	RO	124	Women 18-68								6.5
												(15.5)

* HSV1 or HSV2; ** HAV, HBV or HCV; RO-Romania; HU—Hungary; BG—Bulgaria; SK—Slovakia. Figures in brackets indicate prevalence among non-Roma surveyed. Empty cells indicate no data.

**Table 3:** Parasitic disease (per cent).

First Author	Pub. Year	Country	No. of Roma	Target Group	Trichomonas	Microsporidia	P. h. Capitis and Scabies	Cryptosporidium	Toxocara	Helminths	T. Gondii
Kabakchieva E	2006	BG	296	Young Roma men	8.7						

Halánová M	2013	SK	72	Roma children 0–14 years		30.6					

Djurovic D	2014	SRB	59	Hospitalised children 1–2 years			10.0				
							(0.0)				
Hasajová A	2014	SK	53	Children 0–14				11.3			
								(0.0)			
Antolová D	2015	SK	429	HepaMeta subpopulation					22.1		
									(1.0)		
Pipiková J	2017	SK	275	Children						25.8	
										(0.7)	
Štrkolcová G	2017	SK	60	Children 0–17						85.0	
										(23.8)	
Antolová D	2018	SK	429	HepaMeta subpopulation						* see table footer	
Antolová D	2018	SK	420	HepaMeta subpopulation							45.0
											(24.1)
Fecková M	2020	SK	67	Children							20.9
											(7.1)
Fecková M	2020	SK	67	Children					40.3		
									(2.3)		

* 0.5% Trichinella and 0.2% Echinococcus. BG—Bulgaria; SK—Slovakia; SRB—Serbia. Figures in brackets indicate prevalence among non-Roma surveyed. Empty cells indicate no data.

**Table 4:** Bacterial disease (per cent).

First Author	Pub. Year	Country	No. of Roma	Target Group	Chlamydia	Gonorrhoea	Syphilis
Kabakchieva E	2006	BG	296	Young Roma men	8.0	4.5	3.5
		
Gyarmathy VA	2008	HU	50	Convenience sample of volunteers			1.8
							(0.0)
Gyarmathy VA	2009	HU	42	Injecting Drug Users	* see table footer	0.0	* see table footer

Amirkhanian YA	2013	BG	405	Young Roma men	5.2	3.7	0.0
		
Halánová M	2014	SK	208	HepaMeta subpopulation	7.2		
					(5.3)		

* 16.7% (8.3%) positive for either chlamydia or syphilis. BG—Bulgaria; HU—Hungary; SK—Slovakia. Figures in brackets indicate prevalence among non-Roma surveyed. Empty cells indicate no data.

## Supplementary Materials

The following are available online at https://www.mdpi.com/1660-4601/17/20/7632/s1, Table S1: Roma over Europe, S1: Search terms, Table S2: Quality Assessment.
