# Supplementary material for: Roma Health: An Overview of Communicable Diseases in Eastern and Central Europe
Source: Int J Environ Res Public Health. 2020 Oct 20;17(20):7632. doi: 10.3390/ijerph17207632 (PMC7588998; doi:10.3390/ijerph17207632)
Supplement: Supplementary file 1 [file ijerph-17-07632-s001.pdf]

Table S1: Roma over Europe

Country	Total population (World Bank 2010)	Official number (self-declared)	Census year	Minimum estimate	Maximum estimate	Average estimate	Average estimate as a % of total population
Turkey	72752325	4656	1945	500000	5000000	**2750000**	**3.78%**
Romania	21442012	619007	2011	1200000	2500000	**1850000**	**8.63%**
Russian Federation	141750000	205007	2010	450000	1200000	**825000**	**0.58%**
Bulgaria	7543325	325343	2011	700000	800000	**750000**	**9.94%**
Hungary	10008703	190046	2001	500000	1000000	**750000**	**7.49%**
Spain	46081574	No data available		500000	1000000	**750000**	**1.63%**
Serbia (excl. Kosovo)	7292574	108193	2002	400000	800000	**600000**	**8.23%**
Slovakia	5433456	89920	2001	380000	600000	**490000**	**9.02%**
France	64876618	No data available		300000	500000	**400000**	**0.62%**
Ukraine	45870700	47917	2001	120000	400000	**260000**	**0.57%**
United Kingdom	62218761	No data available		150000	300000	**225000**	**0.36%**
Czech Republic	10525090	11718	2001	150000	250000	**200000**	**1.90%**
Macedonia	2060563	53879	2002	134000	260000	**197000**	**9.56%**
Greece	11319048	No data available		50000	300000	**175000**	**1.55%**
Italy	60483521	No data available		120000	180000	**150000**	**0.25%**
Albania	3204284	1261	2001	80000	150000	**115000**	**3.59%**
Republic of Moldova	3562062	12271	2004	14200	200000	**107100**	**3.01%**
Germany	81702329	No data available		70000	140000	**105000**	**0.13%**
Bosnia and Herzegovina	3760149	8864	1991	40000	76000	**58000**	**1.54%**
Portugal	10642841	No data available		34000	70000	**52000**	**0.49%**
Sweden	9379116	No data available		35000	65000	**50000**	**0.53%**
Belarus	9490500	9927	1999	25000	70000	**47500**	**0.50%**
the Netherlands	16612213	No data available		32000	48000	**40000**	**0.24%**
Ireland	4481430	22435	2006	32000	43000	**37500**	**0.84%**
Kosovo	1815000	45745	1991	25000	50000	**37500**	**2.07%**
Austria	8384745	6273	2001	20000	50000	**35000**	**0.42%**
Croatia	4424161	9463	2001	30000	40000	**35000**	**0.79%**
Poland	38187488	12731	2002	15000	50000	**32500**	**0.09%**
**Country**	**Total population (World Bank 2010)**	**Official number (self-declared)**	**Census year**	**Minimum estimate**	**Maximum estimate**	**Average estimate**	**Average estimate as a % of total population**

Belgium	10879159	No data available		20000	40,000	**30000**	**0.28%**
Switzerland	7825243	No data available		25000	35000	**30000**	**0.38%**
Montenegro	631490	8305	2011	15000	25000	**20000**	**3.17%**
Latvia	2242916	8517	2011	9000	16000	**12500**	**0.56%**
Finland	5363624	No data available		10000	12000	**11000**	**0.21%**
Norway	4885240	No data available		4500	15700	**10100**	**0.21%**
Slovenia	2052821	3246	2002	7000	10000	**8500**	**0.41%**
Lithuania	3320656	2571	2001	2000	4000	**3000**	**0.09%**
Denmark	5544139	No data available		1000	4000	**2500**	**0.05%**
Armenia	3092072	50	2004	2000	2000	**2000**	**0.06%**
Azerbaijan	9047932	No data available		2000	2000	**2000**	**0.02%**
Georgia	4452800	1200	1989	1500	2500	**2000**	**0.04%**
Cyprus	1103647	502	1960	1000	1500	**1250**	**0.11%**
Estonia	1339646	584	2009	600	1500	**1050**	**0.08%**
Luxembourg	505831	No data available		100	500	**300**	**0.06%**
Malta	412961	No data available		0	0	**0**	**0.00%**
Iceland	317398	No data available		0	0	**0**	**0.00%**
Andorra	84864	No data available		0	0	**0**	**0.00%**
Liechtenstein	36032	No data available		0	0	**0**	**0.00%**
Monaco	35407	No data available		0	0	**0**	**0.00%**
San Marino	31534	No data available		0	0	**0**	**0.00%**
Total in Europe	**828510000**	**1809631**		**6206900**	**16313700**	**11260300**	**1.36%**
Council of Europe (47)	**817204500**	**1753959**		**6156900**	**16193700**	**11175300**	**1.37%**
European Union (27)	**502087670**	**1292893**		**4338700**	**7985500**	**6162100**	**1.18%**

*The former Yugoslav Republic of Macedonia. Now known as North Macedonia. Based on a document prepared by the Support Team of the Special Representative of the Secretary General of the Council of Europe for Roma Issues. Accessed in July 2020. Source document updated in 2012. Most estimates include both local Roma + Roma-related groups (Sinti, Travellers, etc.) & Roma migrants. Selected countries highlighted in green. Countries organised by Roma population.

Supplementary Material S1: Search terms

(Roma [TW] OR Romany [TW] OR Rom [TW] OR Rrom* [TW] OR Romani [TW] OR Romanie [TW] OR Gypsies [TW] OR Gipsies [TW] OR Gipsy [TW] OR vlach [TW] OR Sinti [TW] OR Sintis [TW] OR Tzigane* [TW]) AND (Romania* [TW] OR Bulgaria* [TW] OR Hungar* [TW] OR Serbia* [TW] OR Slovak* [TW] OR Czech [TW] OR Macedonia* [TW]) AND (Infections [Mesh] OR Communicable Disease* [TW] OR infect* [TW] OR Epidemic* [TW] OR Pandemic* [TW] OR Disease outbreak* [TW] OR STD [TW] OR STDs [TW] OR Sexually transmitted disease* [TW] OR viral [TW] OR virus* [TW] OR measles [TW] OR hepatitis [TW] OR HIV [TW] OR AIDS [TW] OR mumps [TW] OR Influenza [TW] OR rubella [TW] OR Poliomyelitis [TW] OR Polio [TW] OR Rotavirus [TW]

OR Herpes [TW] OR Papillomavirus [TW] OR HPV [TW] OR Cytomegalovirus [TW] OR Zika [TW] OR coronavirus [TW] OR covid-19 [TW] OR covid [TW] OR Sars-cov-2 [TW] OR Tuberculosis [TW] OR TB [TW] OR Malaria [TW] OR Dengue [TW] OR chikungunya [TW] OR Leishmaniasis [TW] OR helminth* [TW] OR Neisseria gonorrhoeae [TW] OR Chlamydia trachomatis [TW] OR Treponema pallidum [TW] OR syphilis [TW] OR Haemophilus ducreyi [TW] OR chancroid [TW] OR Klebsiella granulomatis [TW] OR Calymmatobacterium granulomatis [TW] OR granuloma inguinale [TW] OR donovanosis [TW] OR Trichomon* vaginalis [TW] OR Candida albicans [TW] OR vulvovaginitis [TW])

Table S2: Quality Assessment

First Author	Journal / Book	Pub. Year	Country	Target group	Was the sample frame appropriate to address the target population? [1]	Was the sample size adequate? [2]	Were study participants recruited in an appropriate way? [3]	Were the study subjects and setting described in detail? [4]	Was data analysis conducted with sufficient coverage of the identified sample? [5]	Were valid methods used for the identification of the condition? [6]	Was the condition measured in a standard, reliable way for all participants? [7]	Was there appropriate statistical analysis? [8]	Was the response rate adequate, and if not, was the low response rate managed appropriately? [9]	Quality Score
Cocu M	AIDS Care	2005	RO	Pregnant women	No	Yes	Yes	Yes	Yes	Yes	Yes	Yes	Yes	**8**
Kabakchieva E	Sex Transm Dis	2006	BG	Young Roma men	No	Yes	Yes	Yes	Yes	Yes	Yes	Yes	Yes	**8**
Gyarmathy VA	Cent Eur J Public Health	2008	HU	Convenience sample of volunteers	No	No	No	Yes	Yes	Yes	Yes	Yes	Yes	**6**
Gyarmathy VA	Eur J Public Health	2009	HU	Injecting Drug Users	No	No	Yes	Yes	Yes	Yes	Yes	Yes	Yes	**7**
Amirkhanian YA	J Immigr Minor Health	2013	BG	Only young Roma men	No	Yes	Yes	Yes	Yes	Yes	Yes	Yes	Yes	**8**
Halánová M	Ann Agric Environ Med	2013	SK	Children 0-14	No	No	No	No	Yes	Yes	Yes	Yes	Yes	**5**
Veselíny E	Cent Eur J Public Health	2014	SK	HepaMeta study: Roma as well as non Roma from the same areas and non Roma from areas without Roma	Yes	Yes	Yes	Yes	Yes	Yes	Yes	Yes	Yes	**7**

Halánová M	Cent Eur J Public Health	2014	SK	HepaMeta subpopulation	No	Yes	No	No	Yes	Yes	Yes	Yes	Yes	**6**
Djurovic D	Eur Rev Med Pharmacol Sci	2014	SRB	Hospitalised children 1-2 years	No	No	No	No	Yes	No	No	Yes	Yes	**3**
Hasajová A	Eur J Clin Microbiol Infect Dis	2014	SK	Children 0-14	No	No	Yes	No	Yes	Yes	Yes	Yes	Yes	**6**
Antolová D	Epidemiol Infect	2015	SK	HepaMeta subpopulation	Yes	Yes	No	No	Yes	Yes	Yes	Yes	Yes	**7**
Pipiková J	Public Health	2017	SK	Children	No	Yes	No	No	Yes	Yes	Yes	Yes	Yes	**6**
Štrkolcová G	Parasitol Res	2017	SK	Children 0-17	No	No	No	No	Yes	Yes	Yes	Yes	Yes	**5**
Antolová D	Int J Environ Res Public Health	2018	SK	HepaMeta subpopulation	Yes	Yes	No	No	Yes	Yes	Yes	Yes	Yes	**7**
Antolová D	Int J Environ Res Public Health	2018	SK	HepaMeta subpopulation	Yes	Yes	No	No	Yes	Yes	Yes	Yes	Yes	**7**
Halánová M	Int J Environ Res Public Health	2018	SK	HepaMeta subpopulation	Yes	Yes	No	No	Yes	Yes	Yes	Yes	Yes	**7**
Ilisiu MB	Ann Glob Health	2019	RO	Women 18-68	No	Yes	Yes	Yes	Yes	Yes	Yes	Yes	Yes	**8**
Fecková M	Folia Microbiol (Praha)	2020	SK	Children	No	No	No	Yes	Yes	Yes	Yes	Yes	Yes	**6**

Fecková M	J Infect Public Health	2020	SK	Children	No	No	No	Yes	Yes	Yes	Yes	Yes	Yes	**6**

1 - General Roma population; 2 - Roma subjects ⩾100; 3 - Random probabilistic sampling or whole sample frame included; 4 - Age, gender distribution; 5 - No coverage bias within the sample; 6 - Standardised, objective tests; 7 - Data collected comparably; 8 - No statistical errors in reporting prevalence
